# Finally, PrEP choices! But will clients ever have a choice?

**DOI:** 10.1002/jia2.26505

**Published:** 2025-07-02

**Authors:** Kimberly E. Green, Kenneth Ngure, Robyn Eakle, Nittaya Phanuphak, Jason Reed

**Affiliations:** ^1^ PATH Geneva Switzerland; ^2^ Harvard University Boston Massachusetts USA; ^3^ Jomo Kenyatta University of Agriculture and Technology Nairobi Kenya; ^4^ University of Washington Seattle Washington USA; ^5^ Independent East Hampton New York USA; ^6^ Institute of HIV Research and Innovation Bangkok Thailand; ^7^ Jhpiego Baltimore Maryland USA

1

Ten years after the World Health Organization (WHO) recommended tenofovir disoproxil fumarate‐based oral pre‐exposure prophylaxis (PrEP) as an additional HIV prevention option, the world is, or rather should be, on the cusp of a biomedical HIV prevention choice revolution. Although oral PrEP scale‐up started slow, particularly in low‐ and middle‐income countries, uptake grew exponentially in Africa and elsewhere to 3.5 million people by 2023 [1]. The end of 2024 represented a convergence of excellence in HIV prevention science, large‐scale country and community leadership in designing and delivering differentiated PrEP services, and visionary financing and programmatic commitment by the President's Emergency Fund for AIDS Relief (PEPFAR) and the Global Fund for AIDS, TB and Malaria (GFATM). By December 2024, two additional products, the dapivirine vaginal ring and long‐acting injectable cabotegravir (CAB‐LA), were newly available in 10 and 12 countries, respectively, and the PURPOSE‐1 and ‐2 trials on a longer‐acting injectable, lenacapavir (LEN), reported astounding near‐perfect efficacy in preventing HIV [2].

Why is choice in PrEP products so anticipated? Several studies have measured substantial unmet PrEP need across populations and geographies when oral PrEP was the only option available. Unmet need is inclusive of those that report intention to start PrEP and/or who report risk factors but who remain PrEP naïve; those that discontinue PrEP but report continued need for PrEP; and individuals using oral PrEP but who prefer a different PrEP product type (e.g. a longer‐acting option). These studies—such as PrEP APPEAL in the Asia‐Pacific and a discrete choice study among women and girls in Kenya, Eswatini and South Africa—measured substantial unmet PrEP need among populations surveyed and preference for a long‐acting product over oral PrEP [3, 4, 5]. Their authors theorized that where a choice in PrEP products was on offer, unmet need would be reduced, PrEP uptake and continuation would be increased, and HIV incidence would fall.

As CAB‐LA and the ring were introduced into the PrEP method mix in countries like Brazil, South Africa and the United States, fairly consistent real‐world PrEP uptake trends emerged indicating a pattern of strong preference for long‐acting injectable PrEP (from 68% to 83% of individuals), and more modest preferences for oral PrEP (17–26%), and the ring (under 5% where included as a PrEP option) [6, 7, 8]. The Dynamic Choice HIV Prevention study in Kenya and Uganda found when services were optimized to provide product choice and service flexibility, PrEP uptake more than doubled. Offering a choice of both CAB‐LA and oral PrEP resulted in 70% of participants opting for any biomedical HIV prevention compared to 13% in the standard of care arm—a 56% difference [9]. While in Brazil, 83% of participants opted for CAB‐LA over oral PrEP as part of the ImPrEP CAB study, with 42% of those opting for CAB‐LA reporting no HIV prevention use in the month preceding. PrEP coverage of the CAB‐LA group was 95% compared to 48−58% for the oral PrEP group, and there were no HIV transmissions among those using CAB‐LA, while incidence rates were between 1.0 and 1.5 per 100 person‐years among those opting for oral PrEP [8]. Importantly, PrEP choice may also be cost‐effective ‐ a recent modelling efforts in South Africa found that introducing LEN alongside oral PrEP would be cost‐effective given the combined potential to more rapidly address unmet PrEP need and reduce new HIV acquisitions [10].

There can be no doubt that offering PrEP choice is a highly promising strategy towards expanding prevention coverage and reaching global goals of reduced HIV incidence by 2030 as part of overall efforts to achieve universal health coverage. The challenge is what to do in the context of the current financial crises, where domestic health funding that was already constrained after the COVID pandemic was shocked again by abrupt large‐scale terminations of United States government donor funding for public health and research.

While very difficult decisions are required by country leaders and global health financiers ‐alike, the following combined supply‐ and demand‐side approaches seem essential in enabling the continuity of PrEP access and choice:

Supply‐side:
  1. Advocate for countries to include the financing of PrEP products and service delivery costs as part of renewed national efforts to increase national health accounts through health (excise) taxes, strategic taxation (e.g. on technology purchases/use, travel), subsidy reductions, and as part of health insurance packages.
  2. Leverage learning from other health products that are more advanced in establishing cost‐effectiveness and transitioning to local financing such as contraceptive methods like subcutaneous depot medroxyprogesterone acetate. Part of DMPA‐SC's success has been in a self‐injection option which has resulted in significant cost reductions [12].
  3. Titrate procurement of CAB‐LA and LEN to focus first on countries with the highest gaps in unmet PrEP need, local demand for the products and likelihood for national adoption including through public and private sector markets.
  4. Demand and enable fair pricing from ViiV Healthcare and Gilead Sciences and licensed generic manufacturers through joint donor‐pooled procurement and volume guarentees while fostering regional purchasing power so governments and private sector channels can directly procure at affordable prices.

Demand‐side:
  1. Further integrate PrEP into existing, locally financed public and private sector primary health care services, including as part of family planning, antenatal care, and key population and youth‐centred or led services [11].
  2. Institutionalize differentiated and de‐medicalized PrEP services including those that are community‐ and pharmacy‐led.
  3. Simplify PrEP services, training, monitoring and reporting to reduce cost, increase sustainability.

Ultimately, turning away from PrEP and the need for long‐acting products will result in increased HIV incidence and cost [13]. The global health community was poised to accelerate oral and long‐acting PrEP choice and drive previously elusive declines in new HIV acquisitions. Will we deliver?

## COMPETING INTERESTS

The authors declare no competing interests.

## AUTHORS’ CONTRIBUTIONS

KEG conceived of the presented idea. The first draft was written by KEG, and all other authors revised and edited subsequent drafts. All authors reviewed the final, submitted draft.

## Data Availability

Data sharing is not applicable to this article as no datasets were generated or analysed during the current study.
